# Its Incidence during Recent Years

**Published:** 1918-08-17

**Authors:** 


### SMALL-POX.

### ITS INCIDENCE DURING RECENT YEARS.

IT has been remarked in THE HOSPITAL that, if there be any foundation for the theory that small-pox is prone to become epidemic at periodic intervals, an outbreak is considerably overdue. In point of fact, small-pox epidemics do not recur with any definite periodicity, and no tendency to such recurrence is to be traced in the statistics given in the Report to the Local Government Board upon the Incidence of Small-pox throughout the World in Recent Years, by Dr. R. Bruce Low, recently published.* Small-pox, like many other infectious diseases, becomes epidemic when infection is allowed to become disseminated amongst a population containing a sufficient number of susceptible individuals: it is, however, peculiar in the fact that a means of securing immunity has been known and practised for many years, and the liability of a community to the occurrence of epidemics depends upon the proportion of its individuals which is protected by recent vaccination or re-vaccination. It is to be remembered that the immunity conferred by vaccination is, like that conferred by an attack of the disease, although an "active" one, evanescent; "the protection afforded by primary vaccination against severity of attack is of longer duration than the protection against attack. . . . Protection against attack cannot be relied upon after the lapse of ten years," or sometimes less. Moreover, the danger to a community of "modified" cases of small-pox is often great, for reasons upon which it is unnecessary to enlarge.

Dr. R. Bruce Low's Report, which contains much detailed and very valuable statistical material, embodies much else which is of even greater practical interest. The stories of the widespread epidemic of small-pox throughout Europe which followed upon the war of 1870 and of the Gloucester outbreak of 1897 are re-told, and to each a peculiar interest attaches at the present time, when conditions not dissimilar to those which preceded these occurrences are well known to exist. Stress is laid upon the frequency with which the infection of small-pox has been conveyed by shipping from port to port, as also by tramps from town to town. The latter method of conveyance has been common in this country, and is evidently well known in America. From the *Chicago Bulletin* is quoted: "As the spring approaches the small-pox-infected wandering hobo begins his pilgrimages, and, as the distribution of small-pox requires no exertion on his part, he becomes a disseminator of the infection." Small-pox of unusually benign strain has been prevalent during recent years in various parts of the world—in the United States, Canada, Brazil, Australia, and New Zealand. The identity of the disease with true small-pox has been disputed, and local names have sometimes been applied to the condition, which has, for instance, been called "Amaas" or "Kaffir-pox" in some parts of South Africa. It has been found in the United States that, "while this benign form is the prevailing type, other and more virulent strains of the infection are occasionally imported, and give rise to fatal outbreaks. . . . Even where the strain of infection is benign some of the persons attacked by it, occasionally, under conditions as yet not quite clear, develop a virulent form of the disease." "When a virulent strain has been imported small-pox shows approximately the same degree of fatality in the United States as in other countries, so that the suggestion that the people have acquired a partial immunity against the disease falls to the ground." Frequent reference is, as might be expected, made to vaccination. "It is not easy," says Dr. Bruce Low, "to write of small-pox without making allusion to vaccination"; nor can it be wished that he had attempted to do so, as not the least valuable parts of the Report are the absolute vindication of the efficacy of vaccination contained in it, and the information which it affords as to the application and reception of vaccination and legislation with regard to it in different parts of the world.

Small-pox is not now dreaded in this country. Within our lifetime, when those rendered blind or so scarred by the disease as to be "shocking objects to those who approached them" were constantly to be met with, the disease was seriously regarded; so seriously that inoculation was practised, and the discovery of the protective power of vaccination was greeted as a boon from Heaven. We are too ready to forget that the epidemic which followed upon the upheaval of the Franco-Prussian War, the last great Western European war, cost at a moderate estimate in Europe alone not less than half a million lives; "should a general intensification of Continental small-pox coincide with the end of the present war and the resumption of free communications, the opportunities of introduction of infection into this country will become numerous and serious." It cannot be too clearly realised that small-pox may manifest great virulence, may spread, and may show a high fatality rate, just as in the past, when it has thoroughly gained a footing in an unvaccinated community. It was stated, so recently as in 1907, that from 50,000 to 100,000 persons die annually from small-pox in Persia, whilst several thousands lose their sight. In Valparaiso during the first seven months of 1905 the municipal authorities are stated to have "removed from the streets thirty-five corpses of persons who had died from small-pox and had been abandoned in panic by their relatives after death had taken place." An account of a "fearful epidemic of small-pox" was published in a Viennese paper, the *Morgen Zeitung*, in 1902; this outbreak occurred in the Grecian island of Fourni, and over 2,000 persons of a population of 2,500 were attacked. "Those of the inhabitants who escaped the infec-

* *Reports to the Local Government Board on Public Health and Medical Subjects.* (New Series. No. 117.)

**Small-pox: Its Incidence during Recent Years**—(*cont.*)

tion deserted their relatives, leaving them to die without food or attention; hundreds of corpses lay unburied, as there was no one available to dig the graves. The island at the time was under Turkish rule, and the officials displayed the most callous indifference to the sufferings of the unfortunate islanders."

The city of Gloucester suffered from small-pox in the 'seventies, after the termination of the Franco-Prussian War, in common with many English towns. For the next twenty years or so it was practically free from small-pox, and it was, until about 1886, fairly classed as a "vaccinated town." About that time a local anti-vaccination society was formed: its efforts met with success, and by 1895, when small-pox infection was introduced from an unknown source, it was estimated that there were about 10,000 unvaccinated children in the city. The disease rapidly assumed epidemic form, and the mortality was heavy, especially, of course, amongst the unvaccinated infants and children and amongst adults who had been vaccinated in infancy but never re-vaccinated. The available hospital accommodation was totally inadequate, and "eventually" temporary hospitals with 700 additional beds were erected. Meanwhile, "it was impossible to isolate the majority of the cases." "The business of the town was paralysed, and the tradespeople suffered great pecuniary losses. The inhabitants of the surrounding districts refused to do their shopping in Gloucester, or even come near it. The monetary loss caused to Gloucester City by the epidemic was estimated by the President of the Gloucester Chamber of Commerce at £150,000." The anti-vaccination society suffered, as it deserved to do, if not so much as it deserved, from the effects of its misdirected zeal, and, in spite of efforts to attribute the epidemic to insanitary conditions in the city, "some of the leading members contracted small-pox, and several died of it; others were vaccinated, the founder of the society and its president, with his whole family, amongst the number."

The popular view of vaccination during the period under review has proved to be remarkably similar in different parts of the world. In the absence of real and present dread of small-pox it has become almost universally a selfish and apathetic one. In Russia "in time of severe epidemics the protection of vaccination and re-vaccination is sought for when alarm arises, but in general it is neglected by the people." In Vienna, in 1915, "the people, who in the absence of small-pox had neglected the protection afforded by vaccination, clamoured loudly for it when small-pox began to spread." "After each small-pox epidemic in Buenos Aires the disease decreases for a time, and, as the population relapses into a state of apathy respecting vaccination, fresh material gradually accumulates, in the shape of unprotected individuals, and ultimately another epidemic develops." The inhabitants of Rio de Janeiro have resisted attempts to enforce vaccination with an energy which proved their undoing in 1904, and will again if they do not learn better.

The anti-vaccination cult is not without its humours—grim and otherwise. Small-pox was prevalent in Salonika in 1909. "At that time the ex-Sultan Abdul Hamid was exiled in Salonika, and, as the danger of his contracting small-pox was regarded as serious, he was vaccinated. In order to assure this august personage that there was little personal danger from the operation, he was first permitted to witness the vaccination of some ten ladies of his harem." At Nice, in 1902, "some trouble was caused to the authorities by the behaviour of an English lady, a Christian Scientist, who went about among the British and American residents in Nice trying to persuade them not to be re-vaccinated. It was suggested that she should be expelled, but, as no legal charge could be brought against her, this suggestion was not carried out. The difficulty was solved in another way, for the lady in question, not having been re-vaccinated, contracted small-pox, of which she quickly died, thus relieving the authorities from any need of further action."

True "anti-vaccinationists" and conscientious objectors to vaccination are rare. The views of these few, by whom we mean those who have taken the trouble to acquire some scientific knowledge of the questions involved and who are still opposed to the principle of vaccination, are accepted or perverted for their own use by other persons, who fall into two classes; firstly, the "cranks" — "political" champions — ever biassed and prepared to pose as objectors to anything which is upheld by constituted law and order and is accepted by the majority; and, secondly, a very large number of people who, having no fear of small-pox before them, prefer to take the easy course of evading or delaying vaccination or re-vaccination until some tangible urgency for it presents itself. Persons of the latter class readily seize the plea of conscientious objection which has been put within such easy reach, supposing (not unreasonably) that the avoidance of vaccination would not have been made so easy if the authorities really attached any great importance to it. Public faith in the efficacy of vaccination is strong, but there is no longer any popular acceptance of the policy that it is necessary for the whole community to be kept in a condition of perpetual immunity by infantile vaccination followed by re-vaccination at intervals throughout life. There is evidently a widespread view that it is no longer the business of the individual to thus protect himself and, incidentally, to protect others; but that it is the affair of the public health authorities to take action as regards small-pox and, if and when necessary, to offer the protection of vaccination or re-vaccination to those exposed to risk of infection. The public, in this country at least, relies upon the efficiency of the public health services and is, moreover, widely possessed of the knowledge that vaccination is protective if speedily performed *after* exposure to small-pox infection. Dr. R. Bruce Low's Report proves, however, that from the point of view of the medical officer of health small-pox still exists as an ever-present danger, and that in time of epidemic its eradication is beset with difficulties, even in a fairly "well-vaccinated" community—a type of community which is becoming increasingly rare.

